# Chondroitin sulfate-functionalized lipid nanoreservoirs: a novel cartilage-targeting approach for intra-articular delivery of cassic acid for osteoarthritis treatment

**DOI:** 10.1080/10717544.2022.2041130

**Published:** 2022-02-21

**Authors:** Heba M. K. Ebada, Maha M. A. Nasra, Rasha A. Nassra, Ossama Y. Abdallah

**Affiliations:** a Central Lab, Faculty of Pharmacy, Damanhour University, Damanhour, Egypt; b Department of Pharmaceutics, Faculty of Pharmacy, Alexandria University, Alexandria, Egypt; c Department of Medical Biochemistery, Faculty of Medicine, Alexandria University, Alexandria, Egypt

**Keywords:** Intra-articular delivery, cartilage targeting, chondroitin sulfate, cassic acid, osteoarthritis

## Abstract

Novel intra-articular nanoreservoirs were implemented employing different cartilage targeting approaches to improve cartilage bioavailability of a chondroprotective drug, cassic acid (CA), for effective amelioration of cartilage deterioration off-targeting CA gastrointestinal disorders. Herein, we compared active cartilage-targeting approach via chondroitin sulfate (CHS) functionalization versus passive targeting using positively charged nanoparticles to target negatively charged cartilage matrix. Firstly, CA integrated nanoreservoirs (CA-NRs) were fabricated based on ionic conjugation between CA and cationic hydrophobic surface modifier octadecylamine (ODA) and were further functionalized with CHS to develop CHS-CA-NRs. Confocal laser microscope was used to visualize the accumulation of nanoparticles into the cartilage tissue. Both targeting approaches promoted CA local cartilage availability and prolonged its residence time. Compared to passive targeted CA-NRs, active targeted CHS-CA-NRs showed higher fluorescence signals in proximity to and inside chondrocytes which lasted for up to 21 days. In MIA-osteoarthritic rats, CHS-CA-NRs showed superior antiosteoarthritic activity, exhibiting highest cartilage repair compared to CA-NRs. Additionally, CHS-CA-NRs significantly inhibited OA inflammatory cytokine, degradation enzyme and oxidative stress and improved cartilage matrix biosynthesis. Conclusively, CHS-CA-NRs improved OA repair showing a superior efficacy for articular cartilage targeting with CHS which could be a potential advance for OA therapy.

## 1. Introduction

Osteoarthritis (OA), a complex disease affecting multiple joint architectures, is characterized by cartilage deterioration and synovium inflammation causing impaired movement of the elderly (van den Bosch 2020; Peng et al. 2021; Jansen & Mastbergen 2022). OA therapy attempts failure arose from a shortage in delivery of antiosteoarthritic drugs to their target site inside the joint, to be able to modify the progression, and treat the structural changes associated with OA while avoiding systemic poor bioavailability and/or associated side effects (Latourte et al. 2020; Morales & Irlandini 2022). Intra-articular (IA) therapy is considered as an appropriate route to localize antiosteoarthritic drugs into the arthritic joint thus minimizing systemic toxicity (Brown et al. 2019; Migliore et al. 2020). Nevertheless, IA delivery systems should overcome the environmental obstacles such as poor cartilage penetration, and rapid clearance that shortens the intra-articular residence time (Liang et al. 2021; Mancipe Castro et al. 2021). Thus, using nanoparticles to target the drug inside the cartilage tissue, the primary site for OA process, is a promising approach (Bedingfield et al. 2021; Mancipe Castro et al. 2021). Cartilage-targeting nanomaterials offer advantages for OA therapy through different strategies for cartilage-targeting; (1) passive targeting that is related to nanoparticles physicochemical properties including size, charge and shape, and (2) active targeting that is governed by decoration of nanoparticles with specific moieties that bind to unique receptors on cartilage matrix or chondrocytes. The most prevalent approach for passive targeting relies on employing positively charged nanoparticles that electrostatically interacted with negatively charged glycosaminoglycans (GAGs), forming the cartilage matrix (Xiao et al. 2021; He et al. 2022). For active cartilage-targeting, it is linked to functionalization of the particles with moieties like chondroitin sulfate (CHS), and type II collagen (Col2)-binding peptides that have potential affinity to specific receptors on the cartilage matrix or on the chondrocytes (Maudens et al. 2018; Brown et al. 2020; Bedingfield et al. 2021).

CHS, a natural anionic glycosaminoglycan, has substantial targeting potential toward human articular cartilage thanks to its interaction with Col2, scavenger receptor CD36 and other receptors on chondrocytes (Sobal et al. 2013, 2016). Therefore, CHS decorated drug delivery carriers promote articular cartilage uptake compared to non-functionalized particles turning the cartilage into a drug reservoir for sustained intra-tissue delivery (Jain et al. 2014; Yin et al. 2017). Additionally, CHS is one of the natural cartilage matrix components and inherently possesses anti-inflammatory and antiosteoarthritic activity making it an approved dietary supplement in USA and Europe (Reginster & Veronese 2021).

Cassic acid, also known as rhein (4,5-dihydroxyanthraquinone-2-carboxylic acid, CA), is a naturaceutical agent belonging to anthraquinones and found in rhubarb (*Cassia reticulate, Rheum palmatum and Rheum undulatum*), and has widespread uses in Chinese medicine (Wang et al. 2020; Li et al. 2021). Since the early 1990s, the diacetyl pro-drug of CA called diacerein (DCN) has received a great clinical appreciation in OA treatment by halting inflammation and repairing the existed cartilage destruction (Almezgagi et al. 2020). CA role in molecular targeting of interleukins inhibition, superoxide anion reduction, and cartilage matrix metalloproteases suppression mechanisms that anticipated in OA pathophysiology was extensively investigated on human OA cartilage explants (Moldovan et al. 2000), murine osteoclasts (Boileau et al. 2008), and bovine chondrocytes (Martin et al. 2003). Moreover, CA could restore the OA catabolic/anabolic imbalance by improving cartilage matrix biosynthesis (Schöngen et al. 1988).

Otherwise, in 2013, oral DCN was recalled according to European Medicines Agency (EMA) owing to its associated gastrointestinal disorders (diarrhea) (Sun et al. 2016). Afterwards, in 2014, EMA announced that DCN's therapeutic benefits outweighed its side effects and specifically recommended the discontinuation of DCN therapy when severe diarrhea occurs (Almezgagi et al. 2020). However, DCN exhibited low bioavailability ascribed to its low aqueous solubility concomitant to malabsorption properties, so different DCN-nanosystems were developed to enhance its bioavailability (Jain et al. 2013; Allam et al. 2017). Yet, few studies reported that IA administration of the pro-drug form (DCN) improved cartilage repair and inhibited inflammation in rats (Jung et al. 2020; Eladawy et al. 2021). However, the extent of DCN intra-articular metabolism hasn’t been investigated which might result in inter-subject variation. In this context, CA represents an ideal chondroprotective drug for OA therapy through IA delivery. So far, no work has assessed the biological efficacy of CA through IA application, despite works that aimed at developing CA loaded PLGA microparticles (Gómez-Gaete et al. 2017) and PLGA nanoparticles (Hu et al. 2020) for IA administration.

Nanotechnology is continuously providing new strategies to expand the opportunity toward drug delivery for OA therapy (Lawson et al. 2021; Rabiei et al. 2021). While several CA loaded nanoparticles were developed, they were not considered efficient for IA application as they lack good encapsulation efficiency and/or continuous sustained drug release for days (Gu & Zheng 2015; Feng et al. 2017; Gómez-Gaete et al. 2018). Therefore, development of CA loaded nanoparticles for IA application was a challenge, as it was featured with low solubility either in water or in most organic solvents especially highly hydrophobic ones (Cheng et al. 2015). Herein, fabricating a lipophilic form of CA based on ionic conjugation with a lipophilic counter-ion (Octadecylamine, ODA) was inspired by CA free carboxylic group. CA pKa values; 4.5 and 8.5 were imputed to its carboxylic and hydroxyl groups, respectively (Petralito et al. 2009). The ionic conjugation approach hold promises in improving the efficiency of lipid based nanosystems to incorporate ionic drugs by increasing their lipophilicity (Asfour et al. 2020; Ismail et al. 2020; Bashyal et al. 2021) and/or in extending the drug release rate by reducing the drug diffusion from the matrix (Malkawi et al. 2020; Ristroph et al. 2021). Here, the ionic conjugate approach was capitalized to engineer CA loaded lipid nanoreservoirs for IA application.

In view of this information, this study aimed for the first time, at developing intra-articular targeted nanoparticles loaded with CA and comparing different cartilage-targeting approaches; passive targeting and active targeting, in terms of in vivo articular cartilage-targeting ability, in vivo residence time, and biological efficacy on arthritic rats to evaluate the optimal approach for OA therapy. In this regard, positively charged CA-NRs were primary developed based on ionic conjugation approach for passive targeting. Afterwards, the selected CA-NRs were functionalized with CHS for active targeting. After more, cartilage-targeting ability and residence time were estimated at predetermined time points post IA injection in healthy and arthritic joints based on CA inherent fluorescence attributes using confocal microscopy. Finally, biological evaluation was performed on MIA-arthritic rats.

## 2. Materials and methods

### 2.1. Materials

Cassic acid was acquired from Nutragreenlife Biotechnology, India. Chondroitin sulfate and Octadecylamine were acquired from Sigma-Aldrich, USA. Compritol 888 ATO, Glyceryl monostearate, Precirol ATO 5, Gelucire 39/01 and Stearic acid were received from Gattefosse, France. Pluronic F-127 was obtained from BASF, Germany. Monoiodoacetate sodium 99% was purchase from ACROS Organics, Belgium.

### 2.2. Apparent lipid solubility

CA apparent solubility in different solid lipids (precirol ATO 5, compritol 888 ATO, glyceryl monostearate, stearic acid, and gelucire 39/01) was assigned using a melt-solubilization technique (Kaur et al. 2017). Briefly, the lipid was added in increments of 0.5 g–5 mg of CA placed in a vial with consistent heating at 5 °C above the melting temperature of respective lipids in a shaker incubator. The least lipid amount that could solubilize CA was determined as CA lipid solubility.

### 2.3. Preparation of cassic acid- octadecylamine (CA-ODA) conjugate

CA-ODA conjugates were prepared at different molar ratios (1:1, 1:2, and 1:3) by adding CA (10 mg) in increments to 10 mL methanolic solution of ODA under stirring until a clear mixture was obtained. The stirring was extended for 2 h at 25 °C and methanol was then vacuum-evaporated.

### 2.4. CA-ODA conjugate characterization

#### 2.4.1. Fourier transform-infrared (FT-IR)

FT-IR spectrum of CA, ODA, and CA-ODA conjugates were scanned between 700–4000 cm^_1^ using FTIR spectrometer (Cary 630, Agilent, USA).

#### 2.4.2. Partition coefficient (P_o/w_) determination

1 mL water (pre-saturated by n-octanol) was added to 1 mL of saturated n-octanol (pre-saturated by water) of CA and CA-ODA conjugates. The samples were agitated at 25 °C for 24 h, and then allowed to stand for 24 h. CA solubility was spectrophotometrically determined at 258 nm. The oil/water partition coefficient (P_o/w_) was calculated from Equation1:

(1) $$P(o/w)=\frac{Cn}{Cw}$$

where Cw and Cn are the solubility of CA in water and n-octanol, respectively.

### 2.5. Preparation of CA-NRs and CHS-CA-NRs

Nanoreservoirs were firstly prepared employing Compritol 888 ATO as a sustained release lipid matrix by melt–emulsification–ultrasonication method (Li et al. 2021). Briefly, 500 mg the lipid was blended with CA-ODA conjugate equivalent to 10 mg CA on a heater of 85 °C. Simultaneously, 300 mg Pluronic F-127 in 5 mL deionized water was heated to 85 °C as well. Afterwards, Pluronic F-127 solution was gently added to and homogeneously dispersed with the drug-lipid melt. The obtained coarse hot o/w emulsion was ultrasonicated at 60% amplitude for 15 min using probe sonicator (Bandelin HD 3100, Germany). Finally, CA-NRs were developed by allowing hot nanoemulsion to cool in an ice bath. After that, 500 µl of CHS solution of different concentrations (0.5–5 g%) was mixed with the selected CA-NRs and stirred at 25 °C for 1 h and then stored at 4 °C. Plain nanoparticles were prepared using the same procedure without addition of drug conjugate to the lipid phase or surface functionalization.

### 2.6. Ca-NRs and CHS-CA-NRs characterization

#### 2.6.1. Particle size (PS), polydispersity index (PDI) and zeta potential (ZP)

PS, PDI and ZP were measured at 25 °C by DLS technique using a Zetasizer (Malvern Instruments Ltd., UK). Dispersions were 40-folds diluted with filtered distilled water. All measurements were performed in triplicate.

#### 2.6.2. Determination of entrapment efficiency (EE%)

EE% was indirectly determined using centrifugal filter tubes (Centricert 1, 100,000 MWCO, Sartorius, Germany) to separate free CA from NRs-associated drug. Then, free CA concentration in the filtrate was spectrophotometerically determined at *λ* max 258 nm after cooling centrifugation for 15 min at 5000 rpm. EE% was calculated from Equation 2:

(2) $$\text{EE}\%=\frac{\text{Total drug amount}-\text{Free drug amount}}{\text{Total drug amount}}\times 100\%$$

#### 2.6.3. Morphological study

CHS-CA-NRs morphology was assessed compared to non-functionalized CA-NRs using TEM (JEM-100S, JOEL Ltd, Japan) after uranyl acetate staining on a carbon- laminated copper grid (Ebada et al. 2021).

#### 2.6.4. Drug release study

Drug release study was implemented using dialysis method. Briefly, 0.5 mL of NRs dispersion was placed into a dialysis bag which was submerged in 15 mL of PBS pH 7.4 at 37 °C in an amber colored glass bottle. The study was executed in triplicates and samples were horizontally shaken at 100 shake/min and 37 °C. Samples (0.5 mL) were withdrawn from the receiver solution at predetermined intervals, replaced with equal volumes of fresh media, and analyzed spectrophotometerically at 258 nm.

### 2.7. *In vivo* studies

#### 2.7.1. Animals and osteoarthritis (OA) model

Adult male Wistar rats (200 ± 20 g) procured from the Experimental Animal Center in Faculty of Medicine, Alexandria University were housed 4–5 rats/cage in pathogen free environment. Animals were kept in standard cages at 23–25 °C and 65% relative humidity with a 12 h light/dark cycle and were freely accessed to water and food. Procedures involving animals and their care were followed the handling guidelines published in UK Directive of 1986; 86/609/EEC and the ethical guidelines of Alexandria University on laboratory animals under ethical approval number 062019518254. In all tests, adequate considerations were adopted to reduce pain or discomfort of animals. To induce OA, under 1-3% isoflurane, an IA injection containing 2 mg of monoiodoacetate (MIA) was administered through the infrapatellar ligament into the knee joint space, in 50 µL saline, via a 26.5-G needle (Naveen et al. 2014; Bryk et al. 2021). Control rats were injected with an equivalent volume of saline.

#### 2.7.2. Cartilage-targeting ability and retention time assessment

The inherent fluorescence attributes of CA (Ebada et al. 2021, 2021) was exploited to determine the residence time and to assess the cartilage-targeting ability of different intra-articular CA-NRs. Fluorescence was quantified in cartilage sections at different intervals in healthy or arthritic knee joints following previous report with some modifications (Sacchetti et al. 2014). Briefly, 50 μL of CHS-CA-NRs, CA-NRs and CA suspension containing 100 µg of CA was unilaterally intra-articularly injected into two rat sets in the left knees: the first set composed of healthy Wistar rats (45 male, 200 ± 20 g) and the second set composed of Wistar rats (45 male, 200 ± 20 g) with OA (2 weeks post MIA single injection). The right knees were kept as control. Three rats from each set and each group were sacrificed by cervical dislocation at day 1, 7, 14, 21 and 28 after treatment.

Following sacrifice, knee joints of animals were taken. The femoro-tibial joint of each rat was removed by cutting halfway through the femur and tibia, samples were then fixed in 10% phosphate-buffered formalin and subsequently decalcified in 5% formic acid for 72 h and inserted in paraffin wax. The cartilage sections were imaged under a confocal microscopy (Leica TCS SP SPE II/DMi 8, Wetzlar, Germany) at excitation wavelength (440 nm) and emission wavelength (520 nm). The confocal microscopy pinhole focus= 137.1 µm, the scan speed = 400 Hz in average, the scan mode is unidirectional in the XYZ axes. Imaging was performed by applying both the fluorescent and DIC (differential interference contrast) modes either individually for each fluorochrome or in a merged pattern (Cogswell & Sheppard 1992). Background fluorescence was determined by imaging a cartilage section of control rat joint not injected with CA formulations and subsequently subtracted from each section before quantification of fluorescence. Three images from different parts of each section were analyzed. For fluorescence quantification, confocal microscopy images were analyzed using Image J software *(*http://ImageJ.nih.gov/ij*).*

#### 2.7.3. *In vivo* efficacy study

Male Wistar rats (200 ± 20 g, *n* = 84) were indiscriminately divided into seven groups. OA was bilaterally induced by single injection of MIA (2 mg/50 µL) in rat knee joints.

Group (1): Normal rats (Negative controls) treated with 50 µL normal saline (*n =* 12).
Group (2): Arthritic rats (Positive controls) treated with 50 µL normal saline (*n =* 12).
Group (3): Arthritic rats treated with 50 µL CA suspension (suspended into deionized water containing 0.5% CMC Na) (100 μg CA, *n =* 12).
Group (4): Arthritic rats treated with 50 µL CA-NRs (F4) (100 μg CA, *n =* 12).
Group (5): Arthritic rats treated with 50 µL CHS-CA-NRs (F8) (100 μg CA, *n =* 12).
Group (6): Arthritic rats treated with 50 µL CHS-NRs (CA-free) (250 μg CHS, *n =* 12).
Group (7): Arthritic rats treated with 50 µL plain NRs (CHS-free, CA-free) (*n =* 12).

**Figure 1.**
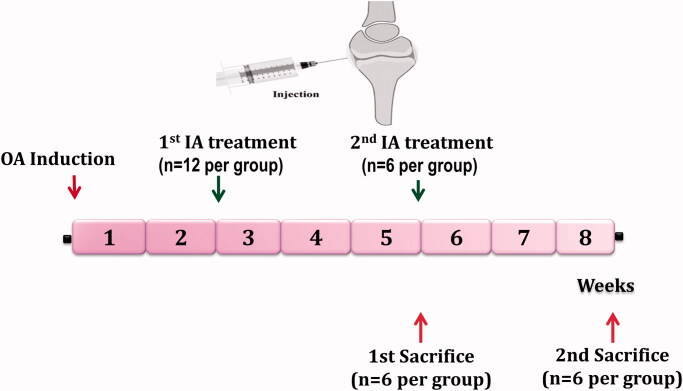
General scheme of the *in vivo* efficacy study. Treatment protocol was over 8 weeks and treatment was started at the end of week 2 and 5 after OA induction. Rats were sacrificed at the end of week 5 and 8, then their joints were evaluated by histological and biochemical analysis.

As illustrated in Figure 1, rats were treated with bilateral IA injection of different formulations at the end of week 2 and 5 after MIA injection for OA induction. Rats were sacrificed for analysis after 5 (*n* = 6) and 8 (*n* = 6) weeks of OA induction. After the first sacrifice, the harvested joints were subjected to homogenization for biochemical assessment in the joint homogenate. After the seconding sacrifice, for each rat, one joint was subjected to histological examination and the second joint was subjected to homogenization for biochemical assessment in the joint homogenate.

##### 2.7.3.1. Histological assessment

The femoro-tibial joint of each rat was removed by cutting halfway through the femur and tibia. Tissue samples were prepared for light microscopy using standard procedures (Gerwin et al. 2010). Briefly, samples were fixed in 10% phosphate-buffered formalin and subsequently decalcified in 5% formic acid for 72 h and inserted in paraffin wax. Sections of femoro-tibial joints were routinely processed and stained with H&E (Hematoxylin and Eosin). Additional set of femoro-tibial joints was stained with toluidine blue. Sections were then subjected to histopathological examination under light microscope. Osteoarthritis Research Society International (OARSI) system was followed to estimate the treated tissue deterioration condition for rats sacrificed after 8 weeks of OA induction (Pritzker et al. 2006; Gerwin et al. 2010).

##### 2.7.3.2. Biochemical analysis

Rat joints were homogenized according to previously reported procedure by Janssen et al. (Janssen et al. 2016). Inflammatory cytokine; interleukin-1 beta (IL-1β) and oxidative stress markers; malondialdehyde (MDA) and nitric oxide (NO), matrix metalloproteinase −3 (MMP-3) and aggrecan levels in joint homogenate were estimated as markers of regeneration capacity (dos Santos Duarte Lana & Rodrigues 2019). IL-1β, MMP-3 and aggrecan were determined using commercial enzyme linked immune sorbent assay (ELISA) kits following the manufacturer's directives (MyBioSource catalog # MBS82501, MBS729026 and MBS721531 respectively). MDA and NO levels were colorimetrically estimated using commercial kits (Biodiagnostic, Egypt) following the manufacturer’s protocol.

##### 2.7.3.3. Statistical analysis

Statistics of the in vitro studies results were accomplished using Student’s t-test with significance level at *p* < .05 (GraphPad Prism 6, USA). Statistical analysis for in vivo studies results was done using SPSS 20 (SPSS, Chicago. IL). The results were analyzed statistically by one-way analysis of variance ANOVA, followed by a Post Hoc (Tukey) test to compare variables between different groups. Statistical significance was set at *p* ˂ .05. Values were expressed as mean ± SD.

## 3. Results and discussion

CA low oral bioavailability along with substantial side effects resulted in unmet clinical need for OA therapy. Thus, in our previous work, the local transdermal route was exploited to deliver CA to the arthritic joint, which has proven significant amelioration in cartilage degeneration, overcoming low bioavailability and side effects (Ebada et al. 2021). Herein, for the first time, cartilage-targeted nanocarriers loaded with chondroprotective CA were tailored and functionalized with CHS for IA injection and evaluated versus non-functionalized ones. Given that cartilage tissue is the primary site affected by OA and it is CAs’ target, the effectiveness of different cartilage-targeting strategies was evaluated according to cartilage biodistribution and retention, and the extent of these outcomes on OA repair was assessed. Moreover, solid lipid-based nanoparticles were employed as potential carriers for sustained drug release as they offer several advantages: first, they are composed of safe, biocompatible and biodegradable ingredients. Besides, their surface can be functionalized with target-specific ligands (Gad et al. 2022). Additionally, for their preparation neither organic solvents nor expensive excipients are needed as in the case of polymeric nanoparticles (Paliwal et al. 2020). One of the crucial factors to develop appropriate lipid nanosystems with good drug loading capacity and sustained release, is the drug lipid solubility. However, upon screening different lipids for solubilization, CA exhibited poor lipid solubility where 5 mg CA needed >5 g of lipid for CA solubilization. This observation was harmonious with CA physicochemical properties as CA shows poor solubility in highly hydrophobic solvents (Cheng et al. 2015).

### 3.1. Preparation and characterization of CA-ODA conjugate

**Figure 2.**
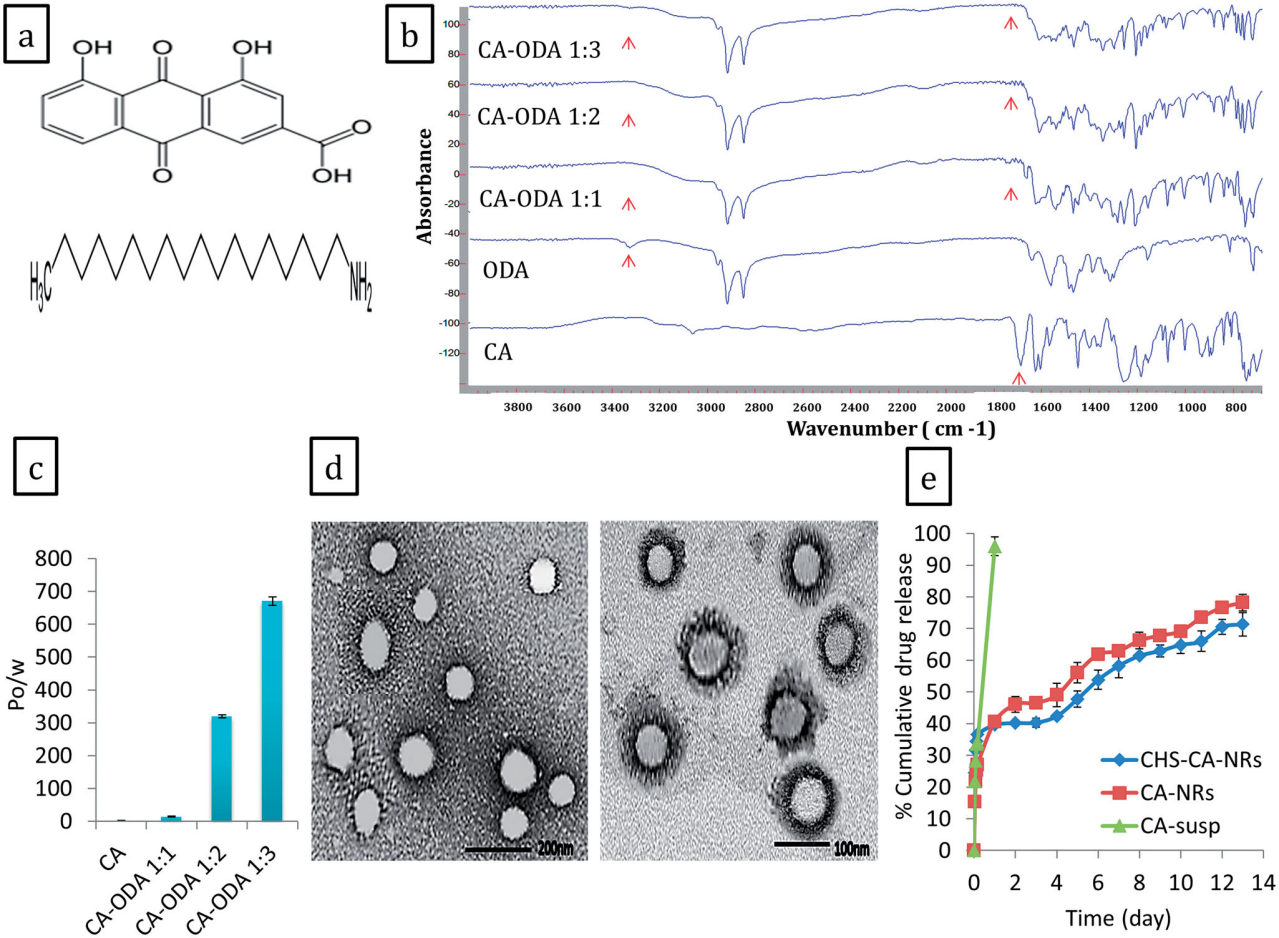
The chemical structures of CA and ODA (a), IR spectra (b) and Partition coefficient (c) of CA, ODA and CA-ODA conjugates (1:1, 1:2, and 1:3). TEM images of CHS-CA-NRs (right) and CA-NRs (left) (d). Release profiles of CA from CA suspension, CA-NRs and CHS-CA-NRs in PBS pH 7.4 at 37°C (e).

For modifying CA lipophilicity without chemical alteration, octadecylamine (ODA) was selected as a lipophilic primary alkyl amine to develop a lipophilic form of CA based on ionic conjugation. The chemical structures of CA and ODA were depicted in Figure 2(a). CA-ODA conjugates formation were verified by FTIR and partition coefficient. FTIR spectra of CA-ODA conjugates manifested an absence of CA peak at 1680 cm^−1^ which was corresponding to C = O stretch of COOH group as illustrated in Figure 2(b). Additionally, the peak of amine group of ODA (3328 cm^−1^) has been diminished in the developed CA-ODA conjugate. Changes in the FTIR spectroscopy indicated the interaction between the amine group of ODA and the carboxyl group of CA, and confirming the formation of CA-ODA conjugate (Jalil et al. 2020; Rodrigues et al. 2020). Additionally, for partition coefficient, compared to CA, CA-ODA conjugates exhibited significant improvement in lipophilicity (Figure 2(c)). In particular, CA-ODA conjugate 1:3 showed high significant improvement (*p* ˂ .0001) where P_o/w_ value was approximately 296 times greater than that of CA (P_o/w_ = 2.26 ± 0.087) confirming the higher lipophilic attribute of CA-ODA conjugate 1:3.

FTIR results and the enhanced oil/water partition could be explained by the electrostatic interaction between CA and ODA that masked their polar groups in addition to the long fatty acid chain of the ODA.

### 3.2. Preparation and characterization of CA-NRs and CHS-CA-NRs

Given CA physicochemical properties, tailoring of lipid NRs loaded with free CA was handicapped by its poor lipophilicity, higher melting point and low drug solubility in most organic solvents which hindered their use during preparation. Therefore, the developed lipophilic CA-ODA conjugates were incorporated into Compritol ATO 888 lipid matrix to develop CA-NRs and afterwards the selected NRs was functionalized with CHS using serial CHS concentrations. CA-NRs loaded with CA-ODA 1:3 conjugate was selected to be functionalized with CHS based on its high drug encapsulation efficiency, controlled release, high positive surface charge that enable functionalization. Composition and characterization of the developed NRs were summarized in Supplemental Table 1.

#### 3.2.1. Particle size (PS), polydispersity index (PDI) and zeta potential (ZP)

The developed NRs laid into the nano-range, as illustrated in Supplemental Table 1. Moreover, there was a gradual increase in PS and PDI together with a corresponding increase in CHS concentration which might be imputed to heterogeneous attachment of CHS on the NRs surface and partial neutralization of the surface charge leading to some particles aggregation. Further increase in CHS concentration resulted in smaller particle size with narrow particles distribution at 0.5% CHS concentration where PS and PDI of **F8** were 156.80 **±** 0.05 nm and 0.27 ± 0.01, respectively. This decrease in PS and PDI values might be imputed to the homogenous distribution of CHS on the NRs surface and also might be most probably explained from the ZP values where the uniform layer of CHS deposited on NRs surface led to high repulsion between particles resulting in particle size decrease.

For the ZP values of CA-NRs, ODA (octadecylamine) altered particles ZP to positive sign compared to ZP of plain NRs (−13.82 ± 0.07 mV) as manifested in Supplemental Table 1. So incorporation of ODA provided CA with the desired lipophilicity to be properly loaded into the NRs lipid matrix and excess ODA acts as surface charge modifier yielding CA-NRs bearing highly positive surface (Castro et al. 2009). Therefore, CA-NRs loaded CA-ODA 1:3 (**F4**) showed the highly positive charged nanoparticles (ZP= 41.83 ± 5.63 mV**)**. For CHS-RA-NRs, Supplemental Table 1 illustrated the progressive decrease in ZP values with CHS concentration increasing, confirming the attachment of negatively charged CHS to the positively charged NRs surface. The final highly negative values after the last two CHS additions (0.5 and 1 g%) (−28.3 ± 6 mV, −30.5 ± 4.03 mV, respectively**)** affirmed the CHS shell existence on CA-NRs surface, this negative charge might arise from the ionized groups of chondroitin sulfate molecules (Luo et al. 2021).

#### 3.2.2. Entrapment efficiency (EE%)

Herein, incorporation of CA-ODA conjugate during NRs preparation enabled CA encapsulation into lipid systems with excellent EE% (99.87 ± 0.23%), as illustrated in supplemental Table 1. This higher EE% might be explained by higher lipid partitioning of CA-ODA conjugate into the lipid matrix. This finding was harmonious with other works that improved the encapsulation of adapalene and breviscapine into lipid nanoparticles based on ionic conjugation with ODA (Li et al. 2013; Jalil et al. 2020).

Upon CHS functionalization, NRs exhibited good EE% ranging from 89.82 ± 0.32% to 99 ± 0.34%. A progressive decrease in EE% was observed upon increasing the CHS conc. which could be explained as follow: as the amount of the added CHS increased, CHS not only interact with the deposited ODA but also compete with CA for ODA involved in CA-ODA conjugate resulting in a slight dissociation in CA-ODA conjugate expelling free CA out of the lipid matrix. This observation might be imputed to the highly negative charge density of CHS (−2) compared to CA (−1) at neutral pH.

Here, this study compared between two different targeting approaches; active targeting using CHS and passive targeting governed with electrostatic interaction between positively charged CA-NRs and negatively charged cartilage matrix components (GAGs) to evaluate the optimal approach in OA therapy. Therefore, **F4** and **F8** were selected as promising passive and active cartilage-targeted NRs, respectively, based on their surface charge, good EE%, acceptable PS and PDI.

#### 3.2.3. Morphological study

In Figure 2(d), TEM image of CHS-CA-NRs (F8) showed spherical, well dispersed and separated nanoparticles with clear deposited dark layer at the surface confirming efficient coating of NRs with CHS. While TEM image of non-functionalized CA-NRs (F4) showed nanoparticles with smooth morphology.

#### 3.2.4. Drug release studies

In Figure 2(e), CA release rate from F8 (CHS-CA-NRs) and F4 (CA-NRs) was significantly slower than that from CA suspension. Ionic-conjugate integrated NRs exhibited biphasic profile with an initial rapid release followed by a sustained slower phase, releasing about 70% of CA over 13 day.

### 3.3. *In vivo* evaluation

In OA animal model, single IA injection of MIA has been vastly used to suppress aerobic glycolysis resulting in chondrocytes death and cartilage structural changes which resemble to OA progress in human (Bryk et al. 2021).

#### 3.3.1. Cartilage-targeting ability and retention time assessment

Cartilage-targeting capability and retention time of different CA formulations in the articular cartilage, as OA hallmark and site of action of CA, was investigated in healthy and OA-bearing rats by trafficking fluorescence under confocal microscope. In this study, rats were intra-articularly injected at day 0 and the joints were harvested at day 1, 7, 14, 21 and 28 post injection, and the cartilages were cut into sections to be observed under a confocal microscope.

**Figure 3.**
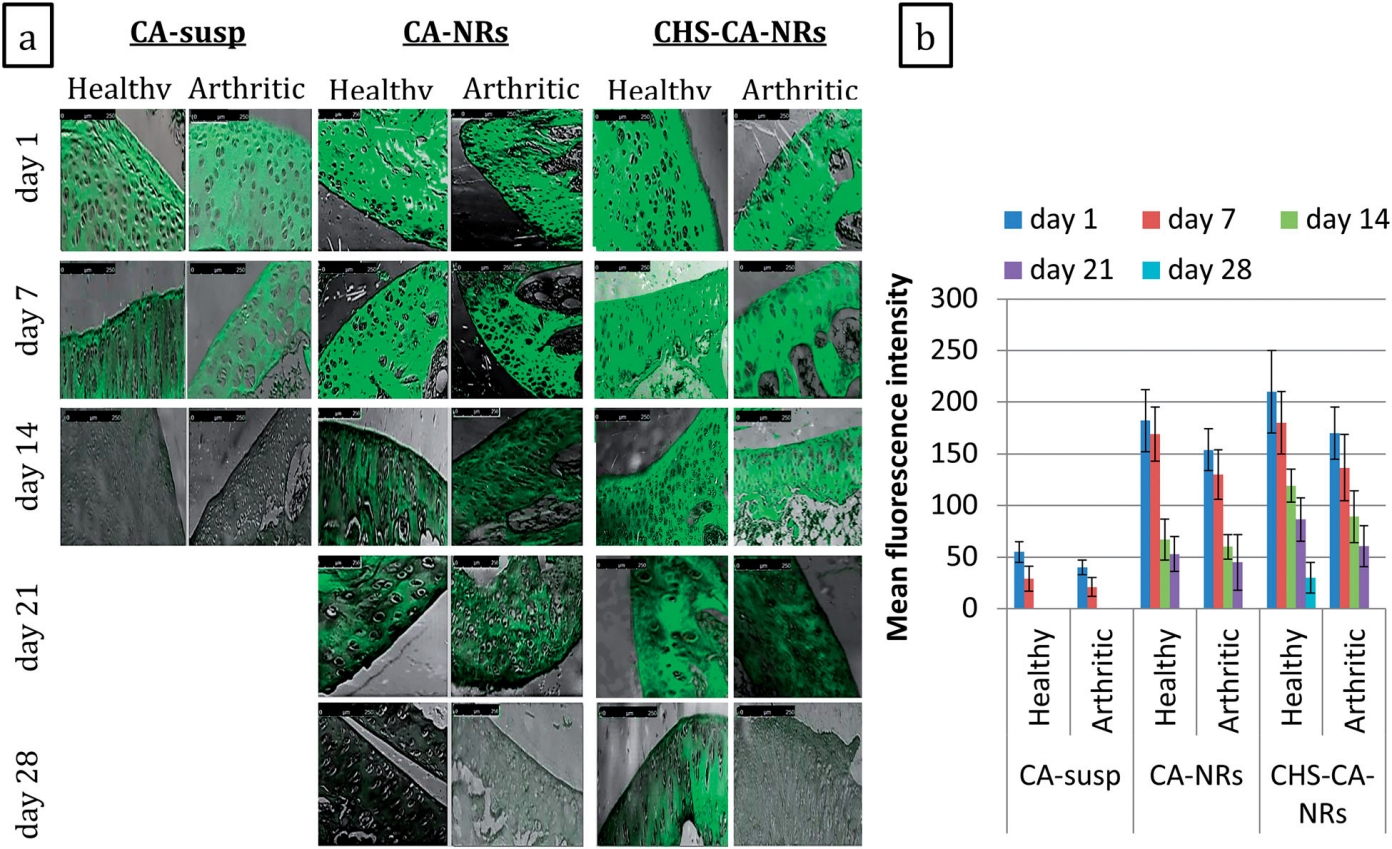
Confocal laser microscopy photos for articular cartilage following single intra-articular injection of different CA formulations containing 100 μg CA/50 μL into the healthy and arthritic knee joints of rats, the fluorescence distribution was observed at day 1, 7, 14, 21 and 28 after administration, magnification × 10 (a). The relative mean fluorescence intensities were determined in articular cartilage of different groups at day 1, 7, 14, 21 and 28 after administration and shown as mean ± SD (*n* = 3) (b).

The cartilage treated with CHS-CA-NRs, either healthy or arthritic groups, revealed the strongest fluorescent green signals (Figure 3(a)). CHS-CA-NRs showed a highly significant increase in fluorescence intensity (210 ± 40 and 170 ± 25 for healthy and arthritic groups, respectively) compared to CA suspension (55 ± 10 and 40 ± 7 for healthy and arthritic groups, respectively) (*p* ˂ .001) after day 1 (Figure 3(b)). Additionally, CHS coating weighed super-fluorescence intensities over that was governed by the electrostatic interaction between positively charged CA-NRs and highly negatively charged cartilage matrix (Figure 3(b)).

**Figure 4.**
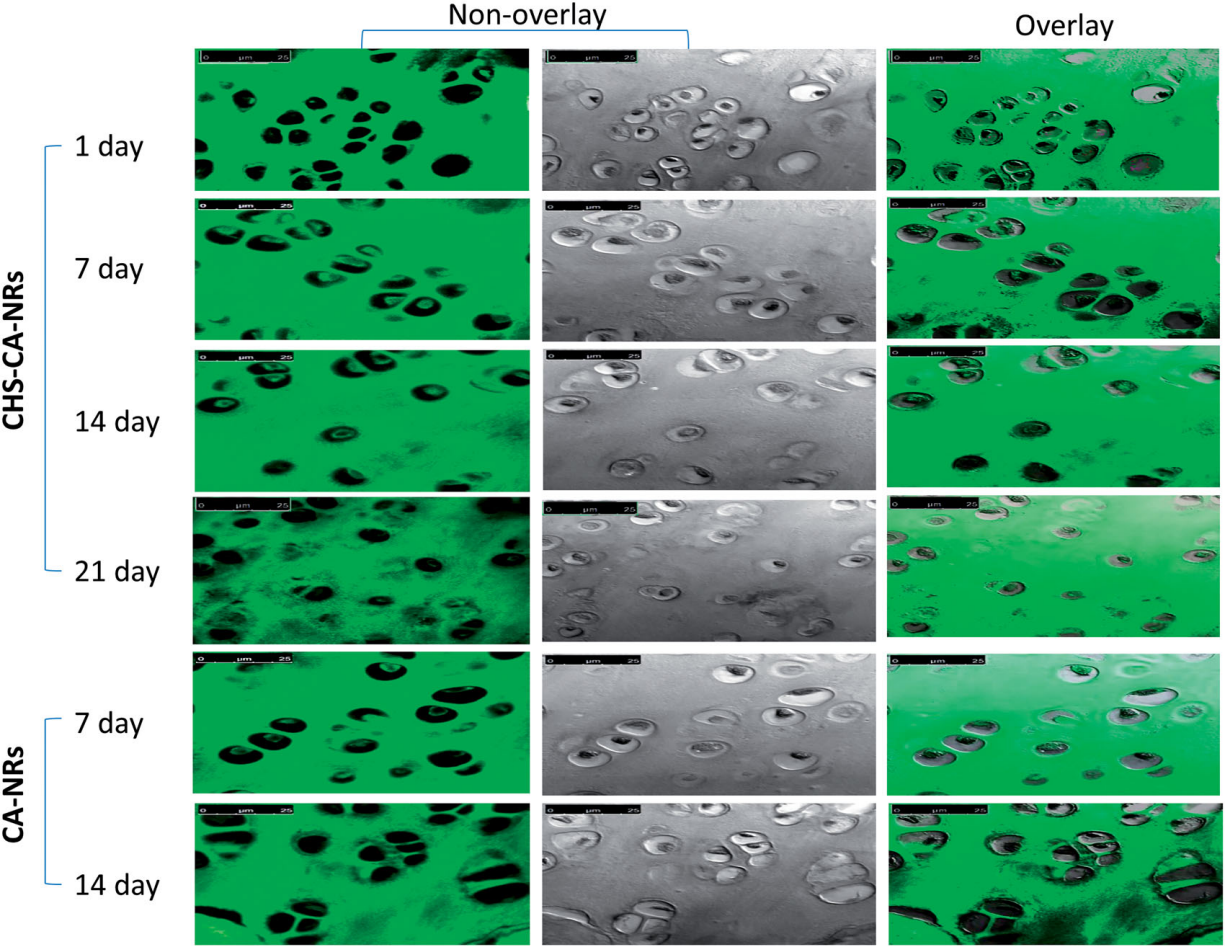
Confocal laser microscopy photos for articular cartilage received CHS-CA-NRs and CA-NRs, showing strong fluorescent signals in the cytoplasm of chondrocytes with CHA-CA-NRs injection over 21 days while little fluorescent signals were observed inside chondrocytes with CA-NRs injection over 14 days, magnification × 63.

Regarding intra-chondrocytes delivery, amid the arthritic groups, the highest fluorescent signals were detected with CHS-CA-NRs at 7 days post injection, and decreased until 21 days (Figure 4). Meanwhile, for CA-NRs, only mild fluorescent signals were detected inside the chondrocytes at 7 days of injection. This result suggested that CHS-CA-NRs specifically facilitated CA delivery and retention in the cartilage and maintained high local concentration in the cartilage for 21 days from injection. These results declared that CHS enabled the nanoparticles to sneak into the cartilage matrix through its nature as a main component of the cartilage matrix. These findings were congruent with previous in vitro, ex vivo and in vivo studies that reported the specific uptake of CHS in human articular cartilage which was most probably due to interaction with Col2 and CD36 and other receptors on chondrocytes, which possess a high affinity for CHS (Sobal et al. 2008, 2013, 2016).

Herein, OA pathology non-significantly affected the distribution profile of intra-articularly CA formulations within the cartilage sections. Nevertheless, in general healthy groups showed more fluorescence compared to arthritic groups at the same time interval for the same formula however the fluorescence of CHS-CA-NRs > CA-NRs > CA suspension. These findings were harmonious with Sacchetti et al. (Sacchetti et al. 2014), while they disagreed with other studies that revealed that arthritis pathological features might affect drug clearance from the joint by inflammation and related increase in vascular permeability, and lymphatic function as well (Pradal et al. 2016).

#### 3.3.2. *In vivo* efficacy study

##### 3.3.2.1. Histological assessment

**Figure 5.**
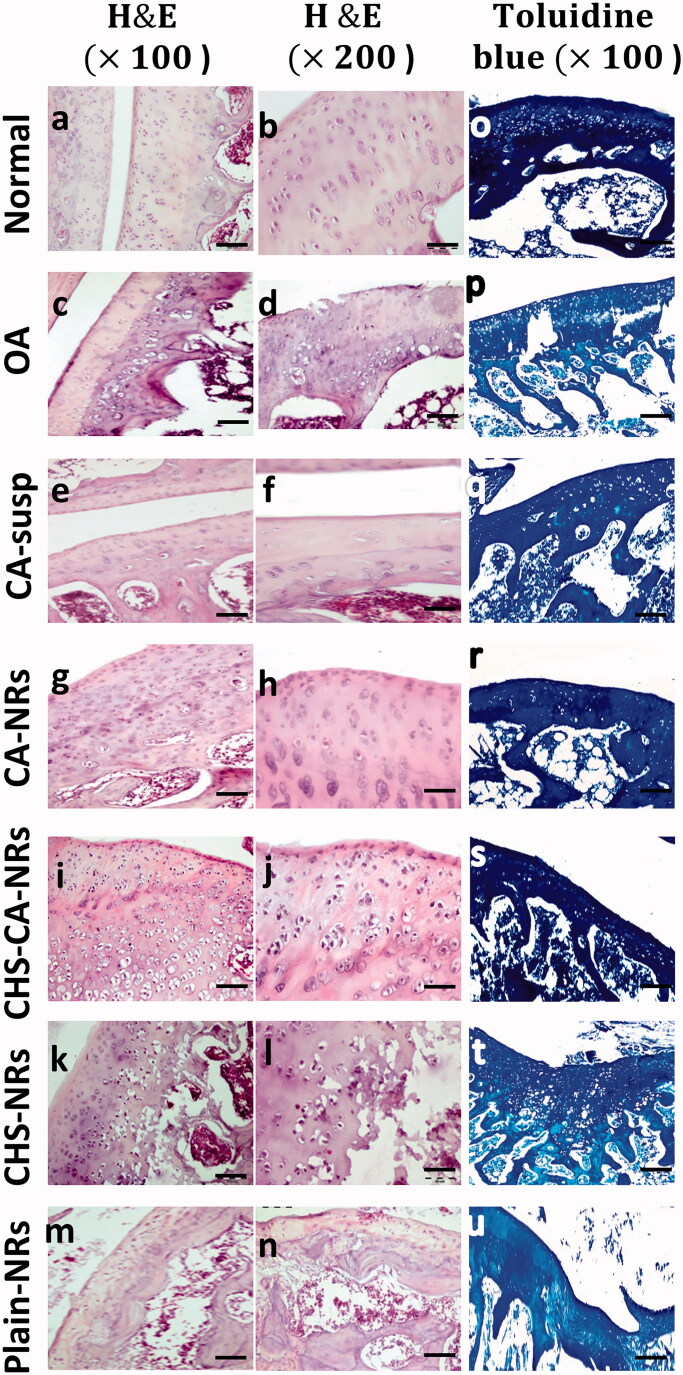
Histological analysis of cartilage sections stained with H&E (a–n) and toluidine blue (o–u) after 8 weeks of OA induction.

**Figure 6.**
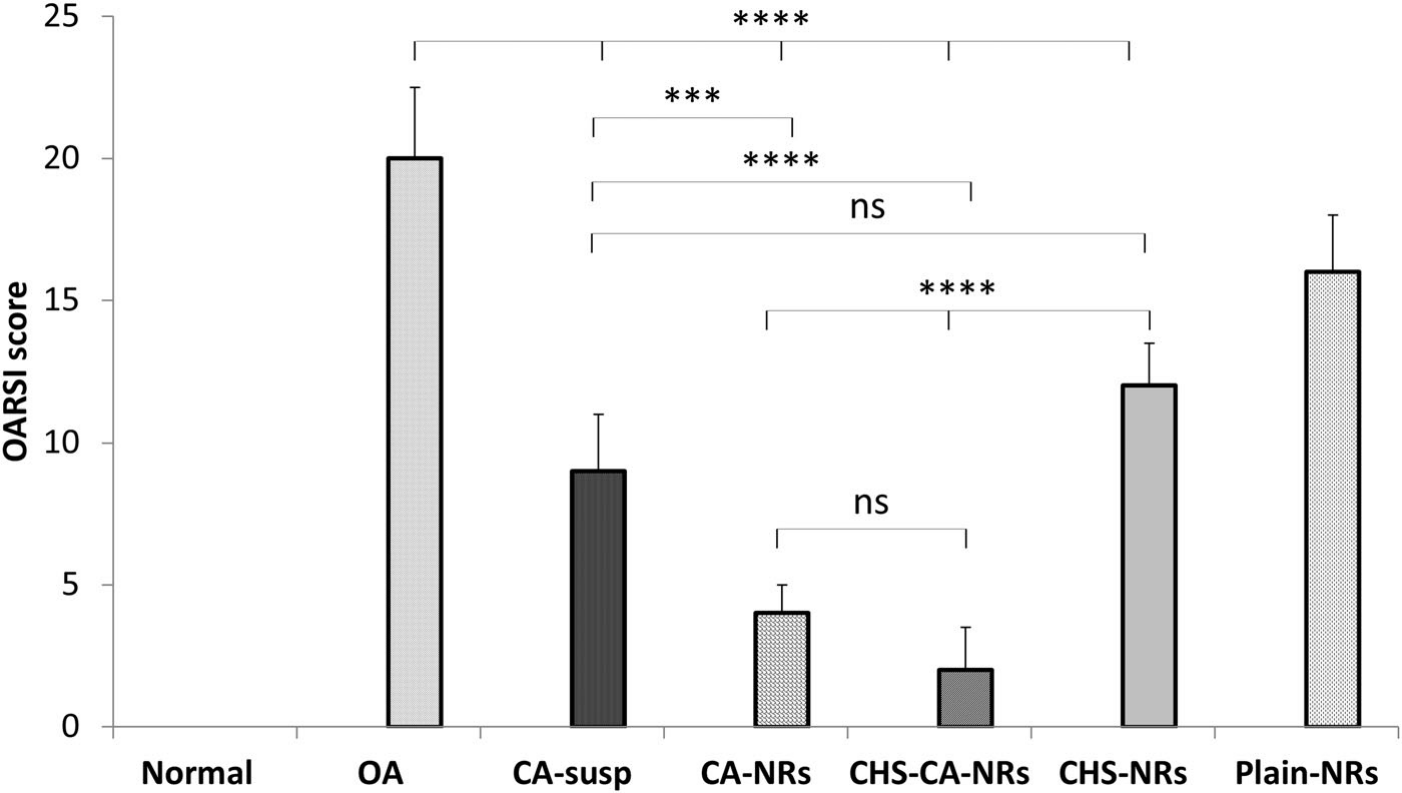
OARSI score of different studied groups after 8 weeks of OA induction. *: *p* < .05, **: *p* < .01, ***: *p* < .001, ****: *p* < .0001 and ns: non-significant.

Histological examination of H&E stained femoro-tibial joint sections in different treated groups at the end of week 8 after OA induction revealed an improvement of cartilage histopathological features in CA-treated groups frustrating the extensive cartilage degeneration Figure 5(a–j). Interestingly, the progression of OA was ameliorated at the greatest level in CHS-CA-NRs group, as evidenced by reduced cartilage degradation (Figure 5(i,j)), more toluidine staining (Figure 5(s)), and the lowest OARSI score (2 ± 1.5) among the treated groups with OA showing non-significant difference compared to normal group (*p* = 0.3962) (Figure 6). While CA-NRs group (Figure 5(g,h)) showed cartilage with significant lower OARSI score (4 ± 1) compared to OA group and CA suspension group (*p* < .0001 and *p* = .0002, respectively) but still significant higher compared to normal group (*p* = .0037).

In comparison to OA group, CHS-NRs group (Figure 5(k,l)) showed significant reduction in OARSI score (12 ± 1.5) but still also significantly higher than normal control, CA-NRs and CHS-CA-NRs groups (*p* < .0001).

##### 3.3.2.2. Biochemical analysis

###### 3.3.2.2.1. Assessment of inflammatory cytokines (IL-1β)

**Figure 7.**
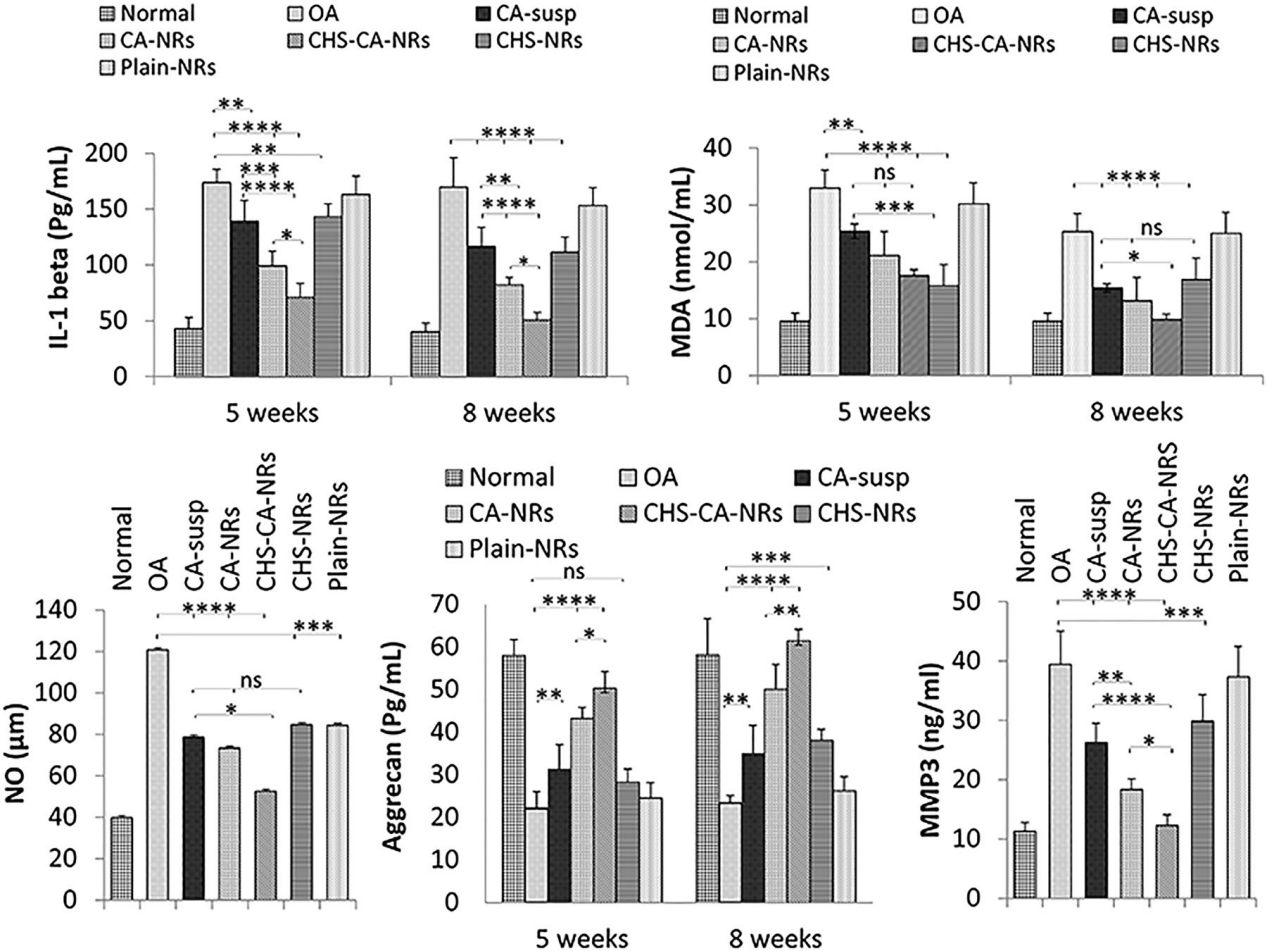
Comparison between the different studied groups according to IL–1 beta (pg/ml) (a), MDA (nmol/ml) (b), NO (µm) (c), Aggrecan level (pg/mL) (d) and MMP3 (ng/mL) (e) in joint homogenate. The data are reported as the mean ± SD (*n* = 6). *: *p* < .05, **: *p* < .01, ***: *p* < .001, ****: *p* < .0001 and ns: non-significant.

At 5 and 8 weeks intervals, the most significant improvement was observed in CHS-CA-NRs treated group (Figure 7(a)) which reached at 8 weeks interval to a non-significant inflammatory level compared to the normal control level [*p* = 0.9047]. While CA-NRs showed higher significant inhibition in inflammatory level compared to CA suspension and CHS-NRs (*p* = .0039 and *p* = .0306, respectively at 8 weeks interval), it exhibited significant lower anti-inflammatory effect compared to CHS-CA-NRs (*p* = .0189). These results indicated the accelerated anti-inflammatory effect of CA in presence of CHS in OA rats.

###### 3.3.2.2.2. Assessment of oxidative stress

The effect of different studied intra-articular formulations on knee homogenate levels of two different oxidative stress markers (malondialdehyde (MDA) and nitric oxide (NO)) was investigated. As seen in Figure 7(b), statistically significant lower levels of MDA were obtained when arthritic rats received intra-articular CA formulations where CHS-CA-NRs manifested the highest antioxidant activity reaching MDA expression close to the normal control level at the end of week 8 interval after OA induction (9.8 ± 0.17 and 9.6 ± 0.0 nmol/mL, respectively). For CHS-NRs, an initially highest significant decrease in MDA level at 5 weeks interval (15.8 ± 1.06 nmol/mL) was observed compared to OA group and almost maintained at 8 weeks interval (16.9 ± 3.74 nmol/mL).

Nitric oxide (NO) is a disruptive agent responsible for OA process progress by mediating the expression of inflammatory cytokines, which inhibits the synthesis of collagen and proteoglycans. The expression of NO was markedly increased in OA articular cartilage and synovium (Leonidou et al. 2018). In the present study, NO level was measured at the end of the experiment period to assess the progress in oxidative stress in joint region.

Figure 7(c) depicted the significant reduction in NO level for all CA treated groups compared to CHS-NRs and plain NRs groups exploiting the potential antioxidant activity of CA in in vivo OA model. Meanwhile, CHS-CA-NRs group showed the highest suppressing effect compared to CA-NRs and CA suspension groups where NO levels were 52.63 ± 9.26, 73.36 ± 11.21 and 78.86 ± 11.50 μM, respectively.

###### 3.3.2.2.3. Assessment of cartilage degradation/synthesis

Regarding aggrecan joint homogenate level as shown in Figure 7(d), the mean level was significantly decreased in OA group compared to the normal control group, while it was significantly increased in all treated groups compared to OA group at 8 weeks interval. CHS-CA-NRs showed the highest elevation in aggrecan level exhibiting significant improvement compared to either CA-NRs (*p* = .0471 and *p* = .0071 at 5 and 8 week intervals, respectively) or CHS-NRs (*p* < .0001 at both intervals).

For MMP-3 level in joint homogenate (Figure 7(e)), CHS-CA-NRs were capable of significantly decreasing the levels of MMP-3 produced by chondrocytes compared to OA group level and all other treated groups (*p* < .0001 compared to CA-susp and CHS-NRs) (*p* = .0354 compared to CA-NRs). These results suggested the potential synergistic antiosteoarthritic effect of CA in presence of CHS.

Amid the groups treated with different CA formulations, CHS-CA-NRs group revealed the most brilliant improvement in both histological and biochemical evaluation parameters. These findings proved the impact of targeting the articular cartilage along with CHS which may be imputed to a synergistic effect by dual mechanism of action on the pathology of degenerative cartilage and/or to the ability of CHS to accelerate the retention of highest amount of CA as previously confirmed by the highest fluorescence observed within the articular cartilage and inside chondrocytes also. CHS coat resulted in evident cartilage regeneration more than this observed in case of CA-NRs as its positively charged surface enabled prolonged retention of CA within the negatively charged matrix however histopathological photos of arthritic cartilage showed depleted areas of matrix and lowest toluidine blue staining confirming the depletion of highly negatively charged GAGs and thus lowering the targeting efficiency. These results were harmonious with other studies that achieved great success in OA therapy capitalizing the cartilage targeting potential of CHS (Bishnoi et al. 2014; Jain et al. 2014; Yin et al. 2017).

While the proven CHS protecting benefits for cartilage and its ability to preferentially enter the cartilage tissue to relieve OA symptoms (Pelletier et al. 2016), the group treated with CHS-NRs showed only 40% decrease in OARSI score which might be imputed to the slow onset of CHS action where its clinical effect appear after about 3-6 months (Bian et al. 2009). Therefore, a prophylactic treatment against the progression of joint degeneration in OA might be most beneficial (Basalo et al. 2007; Fernández-Martín et al. 2021).

It is worth mentioned that IA injection of CHS-CA-NRs could decrease OARSI score by 90% using only 200 μg/joint over 6 weeks treatment. However, an IA injection of DCN loaded nanoparticles (500 μg/joint) offer 64% reduction in OARSI score after 8 weeks treatment (Jung et al. 2020). Herein, These results declared that cartilage-targeted delivery along with CHS has a great potential success on the delivery of natural antiosteoarthritic drug for local OA therapy.

## 4. Conclusion

In this work, this is the first comparison between active and passive cartilage-targeting strategies using chondroitin sulfate coated nanoreservoirs (CHS-CA-NRs) and cationic nanoreservoirs (CA-NRs), respectively, for optimal OA therapy through IA delivery of cassic acid (CA). Cationic CA-NRs were prepared capitalizing ionic conjugation approach to improve drug encapsulation, extend drug release and alter nanoparticles surface charge into positive charge for targeting purpose and CHS functionalization. Active targeted CHS-CA-NRs showed the superior articular cartilage targeting capability lasting for 21 days in proximity to and inside chondrocytes. Moreover, CHS-CA-NRs showed the highest amelioration of cartilage structural alterations with the lowest OARSI score in arthritic rats. Therefore, CHS-CA-NRs hold promises for OA therapy.

## Supplementary Material

Supplemental Material

## Data Availability

Data supporting the study findings are available from the corresponding author on request.
