# Supplementary material for: Chondroitin sulfate-functionalized lipid nanoreservoirs: a novel cartilage-targeting approach for intra-articular delivery of cassic acid for osteoarthritis treatment
Source: Drug Deliv. 2022 Feb 21;29(1):652–63. doi: 10.1080/10717544.2022.2041130 (PMC8865121; doi:10.1080/10717544.2022.2041130)
Supplement: Supplemental Material [file IDRD_A_2041130_SM0905.docx]

**Supplementary data**

**Chondroitin sulfate-functionalized lipid nanoreservoirs : A novel cartilage –targeting approach for intra-articular delivery of cassic acid for osteoarthritis treatment**

by

Heba MK Ebada^a^, Maha MA Nasra^b^, Rasha A Nassra^c^, Ossama Y Abdallah ^b^

^a^ Central Lab, Faculty of Pharmacy, Damanhour University, Damanhour, Egypt.

^b^ Department of Pharmaceutics, Faculty of Pharmacy, Alexandria University, Alexandria, Egypt.

^c^ Department of Medical Biochemistery, Faculty of Medicine, Alexandria University, Alexandria, Egypt.

**Co-authors contact details**

Maha Mohamed Adel Nasra (maha.nasra@alexu.edu.eg)

Rasha Adel Nassra (rasha.nassra@alexmed.edu.eg)

Ossama Youssef Abdallah ( ossama.youssef@alexu.edu.eg)

***Correspondence:**  Heba Mohamed Khairy Ebada. Central Lab, Faculty of Pharmacy, University of Damanhour. El Gomhouria Street, Damanhour Post Office, P.O.Box 22511, Damanhour, Egypt.

Tel : (+2) 01223444145

Fax: 00203 4873273

E-mail: [heba.m.khiry@gmail.com](mailto:heba.m.khiry@gmail.com),

[heba.ebada@pharm.dmu.edu.eg](mailto:heba.ebada@pharm.dmu.edu.eg)

**Experimental**

**Short term stability study**

NRs formulations were stored in the sealed amber colored glass vials in refrigerator. The formulations were analyzed with respect to particle size, PDI, zeta potential and drug entrapment efficiency over 3 months of storage and compared with fresh formulations.

**Synovium histological assessment**

At the end of week 8 after OA induction, the femoro-tibial joint was removed by cutting halfway through the femur and tibia. Tissue samples were prepared for light microscopy using standard procedures [1]. Brieﬂy, samples were ﬁxed in 10% phosphate-buffered formalin and subsequently decalciﬁed in 5% formic acid for 72h and embedded in paraffin wax. Sections of femoro-tibial joints were routinely processed and stained with H&E. Sections were then subjected to histopathological examination of synovium tissue under light microscope.

**Results and discussion**

**Stability study**

The physical stability of either CA-NRs or CHS-CA-NRs was evaluated by measuring particle size, polydispersity index (PDI), zeta potential (ZP), and EE% at 0, 30, 60 and 90 days in refrigerator. As shown in **Table S1**, both NRs showed no dramatic change in particle size or in PDI. This stability might be attributed to the strong electrostatic repulsion of NRs due to their high surface charge, in addition to the CHS coat providing a steric hindrance around the surface of the CHS-CA-NRs. Furthermore, the EE% of both NRs maintained their high values (> 90%) after 90 days in refrigerator which might be due to the stable lipophilic conjugate CA-ODA 1:3 incorporated into the lipid matrix.

It is important to note that the ZP for CA-NRs was positive due to the incorporation of ODA in the lipid composition increasing the zeta potential values. While zeta potential for CHS functionalized NRs was negative due to the electrostatic adsorption of CHS to NRs. A progressive decrease in zeta potential was observed for both formulations, which was more evident for positively charged CA-NRs when compared to CHS-CA-NRs. ZP of CA-NRs where a decrease from 41.82± 5.60 to 25.36±1.43 was observed after 90 days of storage. It is worthwhile commenting on the subtle different stability behavior of CA-NRs and CHS-CA-NRs regarding to ZP values, the decrease of ZP of CA-NRs might be attributed to that some of CA diffused out over time from lipid core to the external medium and subsequently attached electrostatically to the positively charged surface of NRs as CA in the external phase pH (pH =7) was in an ionized form (pka1= 4.5) resulting in decrease in ZP values and this consequently reflect on the attendant higher EE%.

On the other hand, CHS-CA-NRs showed minor change in ZP which decreased from -28.03± 2.07 to -24.14 ±2.62 after 90 days. This might be attributed to that the dissociated CA upon time diffused out of matrix but still dispersed in the external medium as the deposited highly negatively charged CHS at the surface hindered the interaction between diffused CA and excess ODA on the NRs surface which in a good agreement with the concurrent slight decrease in EE%.

**Table S1:** Stability of the CA-NRs and CHS-CA-NRs in refrigerator for 3 months

| **Parameters** | **CA-NRs**  **Time (day)** | | | | **CHS-CA-NRs**  **Time (day)** | | | |
| --- | --- | --- | --- | --- | --- | --- | --- | --- |
|  | **0** | **30** | **60** | **90** | **0** | **30** | **60** | **90** |
| **PS (nm)** | **142.53 ± 1.47** | **151 ± 0.58** | **153.73±**  **1.15** | **157.16 ± 1.47** | **156.80 ± 0.05** | **141.56±1.25** | **141.24±0.64** | **143.74 ± 0.72** |
| **PDI** | **0.25 ± 0.01** | **0.26 ± 0.01** | **0.21 ± 0.01** | **0.25 ± 0.01** | **0.27± 0.01** | **0.20±**  **0.01** | **0.21±**  **0.01** | **0.23± 0.01** |
| **Zeta potential (mV)** | **41.82 ±5.60** | **24.64± 4.45** | **25.13 ± 3.43** | **25.36 ± 1.43** | **-28.30 ± 2.07** | **-25.52 ±6.73** | **-24.35 ±3.75** | **-24.14 ±2.62** |
| **EE (%)** | **99.87±0.23** | **99.92±0.54** | **99.67±0.43** | **99.43 ± 0.24** | **95.83±0.24** | **92.67±**  **2.21** | **91.41±**  **2.46** | **91.11 ± 3.32** |

**Synovium histological assessment**

While loss and degeneration of articular cartilage is a hallmark of OA, pain and disability in patients with OA result from processes involving multiple tissues that contribute to joint structure and function. The synovium may show significant changes, even before visible cartilage degeneration has occurred, with infiltration of mononuclear cells and thickening of the synovial lining layer. Moreover, synovial inflammation is an important source of pro-inflammatory mediators and plays an important role in the progression of the disease. IL-1β produced by chondrocytes and synovial cells is locally increased in OA and induces a large cascade of events leading to cartilage damage.  Both nitric oxide (NO) and prostaglandin E2 (PGE2) produced in large concentrations in OA joints, impair the proliferation of chondrocytes and synoviocytes and enhance the activity of MMPs. The combination of sensitive imaging modalities and tissue examination has confirmed a high prevalence of synovial inflammation in all stages of OA, with a number of studies demonstrating that synovitis is related to pain, poor function and may even be an independent driver of radiographic OA onset and structural progression. Treating key aspects of synovial inflammation therefore holds great promise for analgesia and also for structure modification. In synovial joints, the synovium seals the synovial cavity and fluid from surrounding tissues. The synovium is responsible for the maintenance of synovial fluid volume and composition, mainly by producing lubricin and hyaluronic acid. Through the synovial fluid, the synovium also aids in chondrocyte nutrition, as articular cartilage has no intrinsic vascular or lymphatic supply.^[2]^

Therefore, synovial inflammation was evaluated by histological assessment of synovium of different groups involved in this study at 8 weeks after MIA injection (OA induction). The inflammation and structural changes in the synovial tissue (fibrosis) were reported in the high dose MIA (1mg/ml) rat model [3, 4].

**Fig. S1** depicted photomicrographs of representative sagittal sections of knee joint showing histopathology of synovial membranes. Histological examination of the sagittal sections of synovial membranes of knee joint of osteoarthritis group revealed extensive edema with widely dispersed limited hyperplasia & proliferation of synoviocytes and diffuse lymphoplasmacytic infiltration (a& b). Administration of different RH treatments revealed various degrees of regenerative changes including hyperplasia and proliferation of synoviocytes, villous formation of the membrane and vascular proliferation that were clearly noticed in group CHS-CA-NRs, CA-NRs and CA suspension, respectively (c-h). However, inflammatory signs including lymphoplasmacytic infiltration and hemorrhagic areas were occasionally seen in CA suspension group (c& d). Excess fibroblastic proliferation was manifested with the administration of CHS-NRs (I& j) and plain NRs groups (k& l).

The histopathologial improvement in synovium inflammation was in agreement with the in vitro efficacy of CA on osteoarthritic human synovium cell line showing anti‐proliferative effects by inhibiting both IL-1beta secretion and function in synovial tissue and enhancing interleukin 1 receptor antagonist (IL1ra) production in addition to decrease NO production as well [5-7].

CHS-CA-NRs group showed highest alleviation of synovium inflammation which might be due to the highest CA retained in joint tissue and/or synergistic effect between CA and CHS.

**
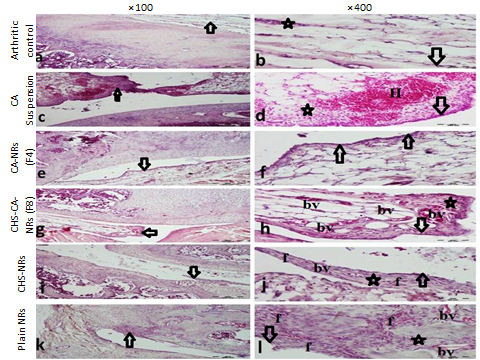
**

**Fig S1:** Photomicrographs of representative sections of knee joint showing histopathology of synovium after 8 weeks of OA induction. Synoviocytes proliferation (arrow), lymphoplasmacytic infiltration (*), hemorrhagic areas (H), blood vessels (bv) and fibroblastic proliferation (f).

[1] N. Gerwin, A.M. Bendele, S. Glasson, C.S. Carlson, The OARSI histopathology initiative – recommendations for histological assessments of osteoarthritis in the rat, Osteoarthritis and Cartilage, 18 (2010) S24-S34.

[2] A. Mathiessen, P.G. Conaghan, Synovitis in osteoarthritis: current understanding with therapeutic implications, Arthritis research & therapy, 19 (2017) 18-18.

[3] T. Hoshino, K. Tsuji, H. Onuma, M. Udo, H. Ueki, M. Akiyama, K. Abula, H. Katagiri, K. Miyatake, T. Watanabe, I. Sekiya, H. Koga, T. Muneta, Persistent synovial inflammation plays important roles in persistent pain development in the rat knee before cartilage degradation reaches the subchondral bone, BMC Musculoskeletal Disorders, 19 (2018) 291.

[4] M. Udo, T. Muneta, K. Tsuji, N. Ozeki, Y. Nakagawa, T. Ohara, R. Saito, K. Yanagisawa, H. Koga, I. Sekiya, Monoiodoacetic acid induces arthritis and synovitis in rats in a dose- and time-dependent manner: proposed model-specific scoring systems, Osteoarthritis and Cartilage, 24 (2016) 1284-1291.

[5] M. Yaron, I. Shirazi, I. Yaron, Anti-interleukin-1 effects of diacerein and rhein in human osteoarthritic synovial tissue and cartilage cultures, Osteoarthritis Cartilage, 7 (1999) 272-280.

[6] F. Legendre, P. Bogdanowicz, G. Martin, F. Domagala, S. Leclercq, J. Pujol, H. Ficheux, Rhein, a diacerhein-derived metabolite, modulates the expression of matrix degrading enzymes and the cell proliferation of articular chondrocytes by inhibiting ERK and JNK-AP-1 dependent pathways, Clinical and experimental rheumatology, 25 (2007) 546.

[7] F. Legendre, A. Heuze, K. Boukerrouche, S. Leclercq, K. Boumediene, P. Galera, F. Domagala, J.P. Pujol, H. Ficheux, Rhein, the metabolite of diacerhein, reduces the proliferation of osteoarthritic chondrocytes and synoviocytes without inducing apoptosis, Scandinavian Journal of Rheumatology, 38 (2009) 104-111.
